# Exploring the Electrochemical Performance of Molybdenum Disulfide Nanoparticles Entrenched in Miscible Poly(methyl methacrylate)-Poly(lactic acid) Blends as Freestanding Electrodes for Supercapacitors

**DOI:** 10.3390/polym16152184

**Published:** 2024-07-31

**Authors:** Bipin S. Chikkatti, Lata S. Kanaki, Ashok M. Sajjan, Nagaraj R. Banapurmath, M. A. Umarfarooq, R. S. Hosmath, Irfan Anjum Badruddin, Amir Ibrahim Ali Arabi, Sarfaraz Kamangar

**Affiliations:** 1 Department of Chemistry, KLE Technological University, Hubballi 580031, India; 2 Centre of Excellence in Material Science, School of Mechanical Engineering, KLE Technological University, Hubballi 580031, India; 3 Mechanical Engineering Department, College of Engineering, King Khalid University, Abha 61421, Saudi Arabia

**Keywords:** polymer membranes, supercapacitors, specific capacitance, energy density, power density

## Abstract

The focus of the study in this article is analyzing the electrochemical properties of molybdenum disulfide on miscible poly(methyl methacrylate)-poly(lactic acid) blends for supercapacitors. The interaction between molybdenum disulfide and miscible poly(methyl methacrylate)-poly(lactic acid) blends, affinity toward water, surface morphology, and mechanical properties are inspected by Fourier transform infrared spectroscopy, water contact angle, scanning electron microscopy, and universal testing machine, respectively. Among the developed membranes, 0.75 wt% of molybdenum disulfide on poly(methyl methacrylate)-poly(lactic acid) shows better electrochemical performances. It exhibits a maximum specific capacitance of 255.5 F g^−1^ at a current density of 1.00 mA g^−1^, maximum energy density of 22.7 Wh kg^−1^, and maximum power density of 360 W kg^−1^. A cycle study reveals 92% capacitance retention after 2500 cycles. The designed supercapacitor device shows a maximum specific capacitance of 1240 μF g^−1^ at a current density of 0.5 μA g^−1^, maximum energy density of 43 μWh kg^−1^, and maximum power density of 700 μW kg^−1^. Flexible membranes of molybdenum disulfide are expected to be a potent combination for supercapacitor applications.

## 1. Introduction

The need for energy is increasing in today’s knowledge-based economies and technologies for storing energy are becoming increasingly crucial [1]. Because of the growing worldwide concern for clean and effective energy sources, such as batteries, supercapacitors, and fuel cells, the development of renewable energy systems is critical [2]. Because of their high power density, prolonged cycle lifespan, and moderate energy density, supercapacitors have received the greatest interest as new types of electrochemical energy-storage devices. Furthermore, supercapacitors are secure and have a low environmental effect [3]. Supercapacitors are gaining popularity because of the growing need for energy storage systems with high specific characteristics and long cycle life. The development of novel materials and storage methods in various applications has led to a lack of broadly agreed terminologies, making it challenging to describe progress in the sector [4]. Supercapacitors are divided into two classes based on how they store charge. To begin, electrical double-layer capacitors store charges at the electrode–electrolyte interface because of ion adsorption. The second type of supercapacitor, pseudocapacitors, stores charges generated by electrochemical processes at the electrode surface [5,6]. To address the constantly increasing demand for flexible portable electronic devices, high-performance and dependable power sources with exceptional flexibility, lightweight, and safety are required [7].

Polylactic acid (PLA) is an environmentally friendly, bioabsorbable, thermoplastic material, crystalline aliphatic polyester derived from sugarcane, maize, and other sources [8]. PLA is now widely used in a variety of sectors because of its beneficial qualities such as excellent mechanical strength, good processing capacity, biocompatibility, ease of molding, good transparency, strong thermal plasticity, and nontoxicity [9,10]. However, because of its high brittleness, low melt viscosity, slow crystallization rate, and low toughness, its uses are limited [11]. One method for overcoming these constraints is to modify PLA through mixing. Polymer blending is a simple industrial procedure that can improve the qualities of current materials by merging the distinct features of different materials [12]. Because of the excellent molecular interactions between poly(methyl methacrylate) (PMMA) and PLA, it is one of the most fascinating thermoplastics to melt-blend with [13]. Because of its physical and mechanical qualities, high Young’s modulus, and hardness ratio, the polymer PMMA is also known as plexiglass, acrylate, or perspex [14]. The PMMA layer may have a role in polar polymer compatibility, such as PLA. Several investigations have shown that PLA mixed with PMMA can form a miscible mixture [15]. PLA/PMMA blends have received a lot of attention due to their synergistic characteristics. When the PMMA level exceeds 50%, the crystallization behavior of PLA is severely hampered and the blends become entirely amorphous [16]. Because PMMA has a higher glass transition temperature than PLA, the miscible addition might increase the temperature resistance of a PLA-matrix in this scenario [17]. Zhang et al. in 2002 reported that Blending PLA with PMMA may be a viable way to produce PLA-based products with better characteristics over PLA alone [18]. 

Layered transition metal dichalcogenides (TMD) have sparked worldwide interest due to their exceptional physical, chemical, electrical, and optical characteristics, which make them ideal candidates for energy storage and conversion [19]. Molybdenum disulfide (MoS_2_) is a usual member of the TMDs family and a suitable material for electrodes for supercapacitors because of its significant theoretical specific capacitance and its stacked structure [20]. MoS_2_ is made up of three atom layers (S-Mo-S) that are held together by van der Waals forces and may be exfoliated to form a single-layer structure using chemical or physical processes [21]. Natural MoS_2_ is semiconducting and has a band gap of 1.2 eV. The monolayer MoS_2_, on the other hand, has a straight bandgap of 1.9 eV, allowing its electrical characteristics to be tuned depending on the application [22]. Some applications, such as devices for storing energy and electrocatalytic processes, require high electrical conductivity, while others, such as sensors, require moderate electrical conductivity. There is currently a lot of curiosity in conductive MoS_2_ for electrode materials because of (a) its excellent electrical conductivity to attain a good current density for the equipment and (b) the potential for ensuring a porous structure, employing this material to aid ionic diffusion of the electrolyte throughout the storage of energy process, which will help to increase the device’s capacity [23]. Chen et al., in 2018, reported that the thermal and mechanical properties of PLA increase as MoS_2_ is added [24]. Dia et al., in 2021, reported that MoS_2_ enhances the electrochemical properties of carbon nanotube/polyaniline [25]. 

Herein, this paper demonstrates an effective strategy for the development of MoS_2_-doped PMMA-PLA membranes as electrodes for supercapacitors. The morphology, structure, and electrochemical nature of the MoS_2_@PMMA-PLA membranes are subsequently analyzed and interpreted in detail. 

## 2. Experiments

### 2.1. Preparation of Membranes

The miscible PMMA (Kemphasol, Mumbai, India)-PLA (Natur Tec India Private Limited, Chennai, India) blend was synthesized by dissolving 8 wt% of PMMA and 2 wt% of PLA in chloroform (Finar Chemicals, Ahmedabad, India) by the solution-casting method [26]. The blend mixture was agitated on a magnetic stirrer at room temperature to obtain a consistent viscous solution. The resulting solution was cast on a clean glass plate and allowed to dry naturally. The completely dried membrane is peeled off and named plain PMMA-PLA. To explore the effect of MoS_2_ nanoparticles (~50 nm, Platonic nanotech, Mahagama, India), different weight percent of MoS_2_ like 0.25, 0.5, 0.75, and 1 wt% were weighed and added to the PMMA-PLA blend solution and named 0.25 wt% MoS_2_@PMMA-PLA, 0.5 wt% MoS_2_@PMMA-PLA, 0.75 wt% MoS_2_@PMMA-PLA, and 1 wt% MoS_2_@PMMA-PLA, respectively. After the addition of MoS_2_, the solutions were sonicated for 30 min and stirred overnight on a magnetic stirrer at room temperature. The resultant homogenous solutions were cast on a clean glass plate and allowed to dry naturally. Fully dried membranes were peeled off and considered for characterization. 

### 2.2. Development of a Supercapacitor Device

**Figure 1:**
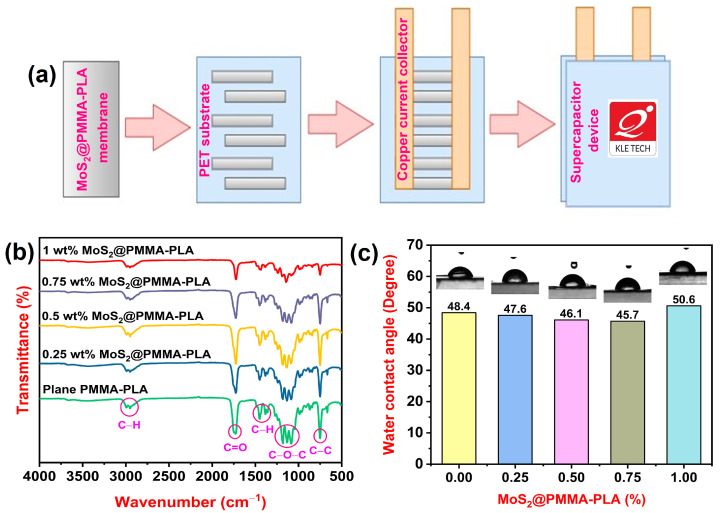
(**a**) Scheme of fabrication of the supercapacitor device; (**b**) FTIR spectra of plain PMMA-PLA and MoS_2_-incorporated PMMA-PLA membranes; (**c**) Water contact angle measurements of plain PMMA-PLA and MoS_2_-incorporated PMMA-PLA membranes.

The supercapacitor device was designed from copper sheet, electrode materials, and PVA (S. D. Fine Chem Limited, Mumbai, India)-H_2_SO_4_ (Spectrum Reagents and Chemicals, Ernakulam, India) gel. The copper sheet was cleaned with acetone and distilled water many times. To prepare the PVA-H_2_SO_4_ gel, 2 g PVA was added into 20 mL of 10 wt% aqueous solution of H_2_SO_4_ and agitated at 60 °C for 3 h. The gel was given time to cool to the ambient temperature before being utilized to construct the gadget. The MoS_2_@PMMA-PLA membrane was made into strips and positioned on a polyethylene terephthalate (PET) sheet. A copper sheet was used as a current collector. PVA-H_2_SO_4_ gel electrolyte was placed in between the membrane strips and allowed to dry completely. Later, the device was sealed using another PET sheet. Figure 1a represents the scheme of fabrication of the device.

### 2.3. Physicochemical Measurements

The Fourier transform infrared spectroscopy (FTIR) technique was executed for all fabricated membranes on the FTIR Spectrometer, PerkinElmer spectrum, Singapore, to confirm the functionalization. The water contact angle (WCA) technique was performed by the Sessile drop technique and a contact angle meter, Kyowa Interface measurement and analysis, Japan, to understand affinity toward water. A scanning electron microscope (SEM) was utilized to analyze surface morphology by Zeiss Ultra 55 Gemini, Graz, Austria, equipped with energy dispersive spectroscopy (EDS), Oxford Instruments X-Max, High Wycombe, UK. Universal testing machine (UTM) DAK system Inc., Thane, India, was utilized to measure the mechanical properties of membranes.

### 2.4. Electrochemical Measurements

A CHI660E electrochemical workstation from CH Instruments, Austin, TX, USA, was used to conduct electrochemical investigations on MoS_2_@PMMA-PLA membranes. In 1 M H_2_SO_4_ aqueous solution, all electrochemical experiments were performed using a two-electrode configuration. At room temperature, electrochemical characterizations such as cyclic voltammetry (CV), electrochemical impedance spectroscopy (EIS), and galvanostatic charge–discharge (GCD) tests were conducted. CV techniques are performed at different scan rates (5 to 100 mV s^−1^) in the potential range of −0.8 to +0.8 V. A frequency range of 1–100 kHz with an amplitude of 5 mV was considered during EIS analysis. Different current densities from 1 to 2.25 mA g^−1^ were used during the GCD technique. The specific capacitance (Csp) of a single membrane electrode using the CV technique is calculated using Equation (1).

Csp = 2∫IdV/m∆VS(1)

where ∫IdV is the area under the CV curve, m is the mass of the electrodes, ∆V is the potential range, and S is the scan rate. The specific capacitance (Csp) of a single membrane electrode using the GCD technique is calculated using Equation (2).

Csp = 4I∆t/m∆V(2)

where I is the discharge current, ∆t is the discharge time, m is the mass of electrodes, and ∆V is the potential range. The energy density (E) of the membrane electrode is calculated using Equation (3).

E = Csp(∆V)^2^/2 (3)

where Csp is specific capacitance and ∆V is the potential range. The power density (P) of the membrane electrode is calculated using Equation (4).

P = E/∆t(4)

where E is energy density and ∆t is the discharge time. Suitable unit conversions are made during calculations [27,28].

## 3. Results

### 3.1. FTIR Study

Figure 1b demonstrates the FTIR spectra of plain PMMA-PLA and MoS_2_-loaded PMMA-PLA membranes. The peaks observed at 2990 cm^−1^ and 2947 cm^−1^ are mainly due to the C-H stretching of both PMMA and PLA [29,30]. The stretching mode of the carbonyl groups showed an absorption band at 1723 cm^−1^. The several bands at 1435 and 1384 cm^−1^ are attributed to C-H deformations. The C–O–C stretching of PMMA and PLA causes peaks at 1193, 1144, and 1091 cm^−1^. The band at 748 cm^−1^ is related to the C-C stretching of blends [31,32]. As the amount of MoS_2_ increases in PMMA-PLA blends, the intensity of the peaks decreases. This is because MoS_2_ can potentially form hydrogen bonds with PMMA-PLA blends. These interactions might weaken the existing hydrogen bonds between PLA and PMMA chains, leading to a decrease in peak intensity associated with those bonds.

### 3.2. WCA Study

Figure 1c illustrates the WCA measurements of fabricated membranes. In general, the lesser the value of the water contact angle, the greater the hydrophilicity nature of the membrane. WCA findings of plain PMMA-PLA, 0.25 wt% MoS_2_@PMMA-PLA, 0.5 wt% MoS_2_@PMMA-PLA, 0.75 wt% MoS_2_@PMMA-PLA, and 1 wt% MoS_2_@PMMA-PLA are 48.4, 47.6, 46.1, 45.1, and 50.6 degrees, respectively. As the content of MoS_2_ in PMMA-PLA blends increases from 0.25 wt% to 0.75 wt%, the WCA values decrease, indicating the hydrophilicity of the membranes increases. This is because MoS_2_ has a layered structure with a large surface area and can increase the surface roughness and porosity of PLA-PMMA blends. This rougher surface can provide more grip for water droplets, leading to a decrease in the contact angle. As the content of MoS_2_ in PMMA-PLA blends increases from 0.75 wt% to 1 wt%, it is observed that the WCA value increases. This is because the casting solution becomes more viscous at this MoS_2_ concentration. As a result, a denser membrane is produced, which contains fewer pores when related to the other PMMA-PLA membranes. Furthermore, porosity and the contact angle are inversely proportional to one another [33].

### 3.3. SEM Study

**Figure 2:**
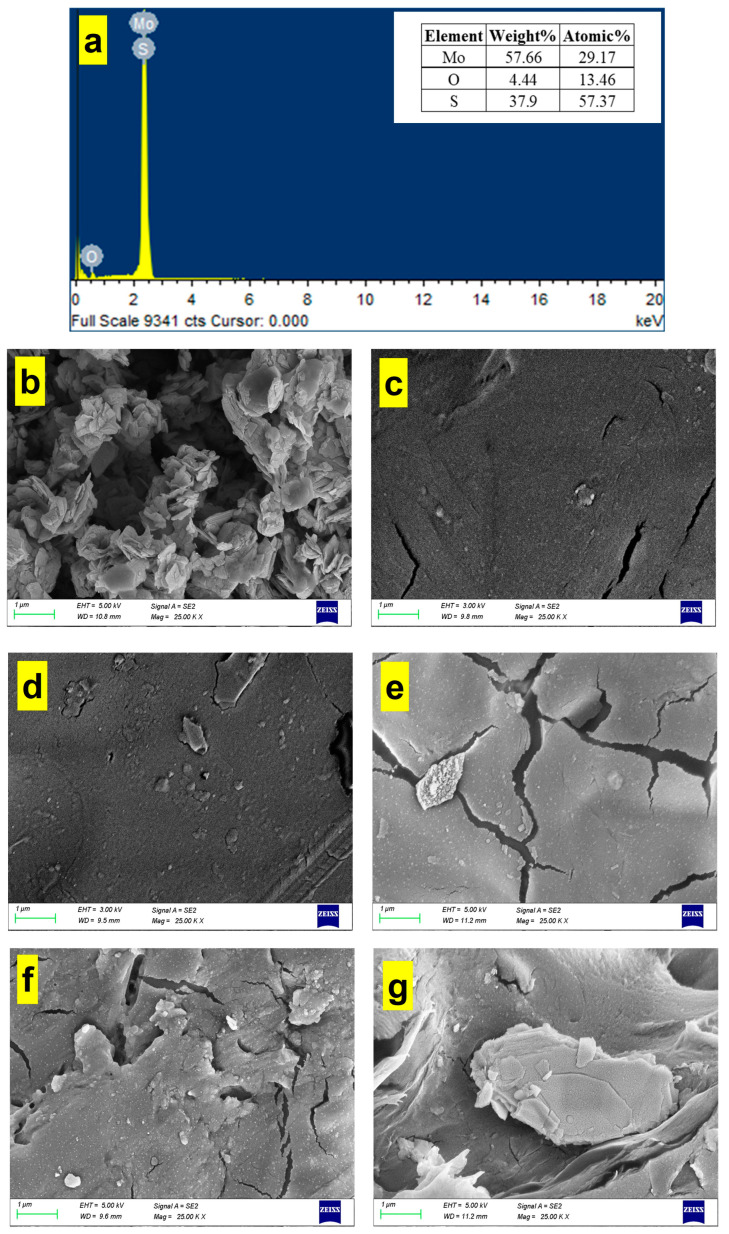
(**a**) EDS analysis of MoS_2_; (**b**) SEM image of MoS_2_ nanoparticle; (**c**–**g**) SEM images of plain PMMA-PLA, 0.25 wt% MoS_2_@PMMA-PLA, 0.5 wt% MoS_2_@PMMA-PLA, 0.75 wt% MoS_2_@PMMA-PLA, and 1 wt% MoS_2_@PMMA-PLA, respectively.

The EDS analysis of MoS_2_ nanoparticles is displayed in Figure 2a, which shows that the nanoparticle is principally composed of molybdenum, oxygen, and sulfur with weight % of 57.66, 4.44, and 37.9%, respectively. Figure 2b displays an SEM image of the MoS_2_ nanoparticle. The SEM image shows a cluster of MoS_2_ nanoparticles, which appear as roughly spherical particles [34]. The surface of the nanoparticles is rough and textured, which is indicative of their crystalline nature. Figure 2c represents an SEM image of the plain PMMA-PLA membrane. No phase separation was observed on the fracture surface of the blend, suggesting that the PMMA-PLA is a miscible blend [35]. By adding MoS_2_, the surface was no longer as smooth as the plain PMMA-PLA. Figure 2d–g displays SEM images of 0.25 wt% MoS_2_@PMMA-PLA, 0.5 wt% MoS_2_@PMMA-PLA, 0.75 wt% MoS_2_@PMMA-PLA, and 1 wt% MoS_2_@PMMA-PLA, respectively. When the amount of MoS_2_@PMMA-PLA was increased from 0.25 to 0.75 wt%, the presence of hydrophilic MoS_2_ in the casting solution accelerated the formation of the microvoid structures and the surface became rough with microcracking. However, the number of macropores was reduced with considerable agglomeration and clustering of the MoS_2_ nanofillers at a higher additive concentration of 1 wt% and the internal structure was more compact. This was because rheology played a leading role in the increase in the viscosity of the casting solution with the addition of MoS_2_, which densified the membrane structure [36].

### 3.4. UTM Study

The mechanical properties of developed membranes carried out at a load speed of 25.0 mm/min are shown in Table 1. Plain PMMA-PLA is quite stiff and brittle. The tensile strength is 11.1 MPa and Young’s modulus is 2130 MPa, whereas elongation at the break is 1.07%. Tensile strength and Young’s modulus rise when the MoS_2_ concentration in PMMA-PLA increases from 0.25 wt% to 0.75 wt% but the elongation break decreases. The mechanical property increases are directly related to the nanofiller dispersion state and nanofiller alignment in the matrix may further strengthen the reinforced effect. The considerable mechanical elevation with such a modest MoS_2_ dosage might be attributed to consistent MoS_2_ dispersion and alignment inside the polymer matrix [37,38]. Furthermore, MoS_2_ can act as a reinforcing filler, providing additional strength and stiffness to the material. As the MoS_2_ concentration in PMMA-PLA increases from 0.75 wt% to 1 wt%, tensile strength and Young’s modulus decrease, whereas elongation break values increase. The mechanical characteristics have worsened because of significant agglomeration and clustering of the MoS_2_ fillers at high loading percentages. Filler agglomeration reduces the interfacial contact area between the MoS_2_ and matrix, reducing the MoS_2_’s capacity to strengthen the composition [39].

### 3.5. CV Study

**Figure 3:**
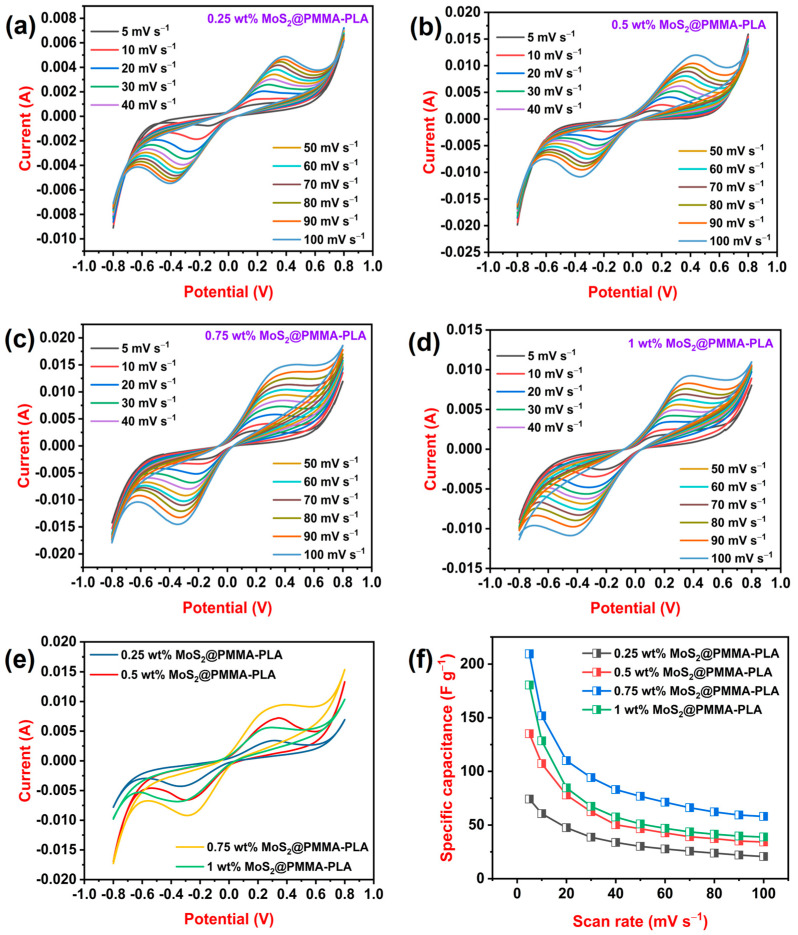
(**a**–**d**) Cyclic voltammograms of 0.25 wt% MoS_2_@PMMA-PLA, 0.5 wt% MoS_2_@PMMA-PLA, 0.75 wt% MoS_2_@PMMA-PLA, and 1 wt% MoS_2_@PMMA-PLA at variable scan rates; (**e**) Cyclic voltammograms of 0.25 wt% MoS_2_@PMMA-PLA, 0.5 wt% MoS_2_@PMMA-PLA, 0.75 wt% MoS_2_@PMMA-PLA, and 1 wt% MoS_2_@PMMA-PLA at scan rate of 50 mV s^−1^; (**f**) Variation in Csp concerning variable scan rate of 0.25 wt% MoS_2_@PMMA-PLA, 0.5 wt% MoS_2_@PMMA-PLA, 0.75 wt% MoS_2_@PMMA-PLA, and 1 wt% MoS_2_@PMMA-PLA membranes.

CV curves of MoS_2_-loaded PMMA-PLA membranes are recorded at variable scan rates. Figure 3a–d displays cyclic voltammograms of 0.25, 0.5, 0.75, and 1 wt%, respectively. As the scan rate rises, the area beneath the curve as well as the peak current increases [40]. This is because, as the scan rate rises, the potential changes faster, which means there is less time for the electroactive species to diffuse toward the electrode surface. This leads to a higher concentration of the species near the electrode surface, resulting in a larger current. All CV curves display redox peaks, which indicate typical pseudocapacitance behavior. This is due to electron insertion and extraction and the Faraday reaction may store the ions in the active regions of the materials. As a result, MoS_2_ is responsible for the faradaic pseudocapacitances and electroconductive properties of the composites. The Faradaic redox reaction, due to the various oxidation states of Mo, and cation intercalation (H^+^) in between the interlayers [41] can be represented as follows:

MoS_2_ + H^+^ + e^−^ ↔ HMoS_2_ (5)

Figure 3e displays the CV curve of all MoS_2_-loaded PMMA-PLA membranes at a fixed scan rate (50 mV s^−1^). From the graph, it is observed that 0.75 wt% of MoS_2_ at the PMMA-PLA membrane showed the highest peak current and area under the curve. As the amount of MoS_2_ increases from 0.25 wt% to 0.75 wt%, the area beneath the curve increases due to the presence of lots of active material, MoS_2_, at the PMMA-PLA membrane. When the amount of MoS_2_ reaches 1 wt%, the area under the curve decreases because of the agglomeration of MoS_2_ nanofiller. The specific capacitance of all MoS2@PMMA-PLA was calculated using Equation (1). Figure 3f illustrates the variation in Csp concerning the scan rates of all fabricated membranes. At a scan rate of 5 mV s^−1^, 0.25 wt% MoS_2_@PMMA-PLA, 0.5 wt% MoS_2_@PMMA-PLA, 0.75 wt% MoS_2_@PMMA-PLA, and 1 wt% MoS_2_@PMMA-PLA displayed a specific capacitance of 74, 135, 209, and 199 F g^−1^, respectively. At all scan rates, 0.75 wt% MoS_2_@PMMA-PLA showed the highest specific capacitance, which is considered an optimized membrane. The material’s specific capacitance falls as the scan rate increases. This is because, at a low scan rate, ions have plenty of time to reach and react with the entire electrode surface, leading to maximum utilization and hence higher Csp. Whereas, at a high scan rate, ion diffusion and reaction are limited, restricting the active area and reducing the amount of charge stored, resulting in a lower Csp.

### 3.6. EIS Study

**Figure 4:**
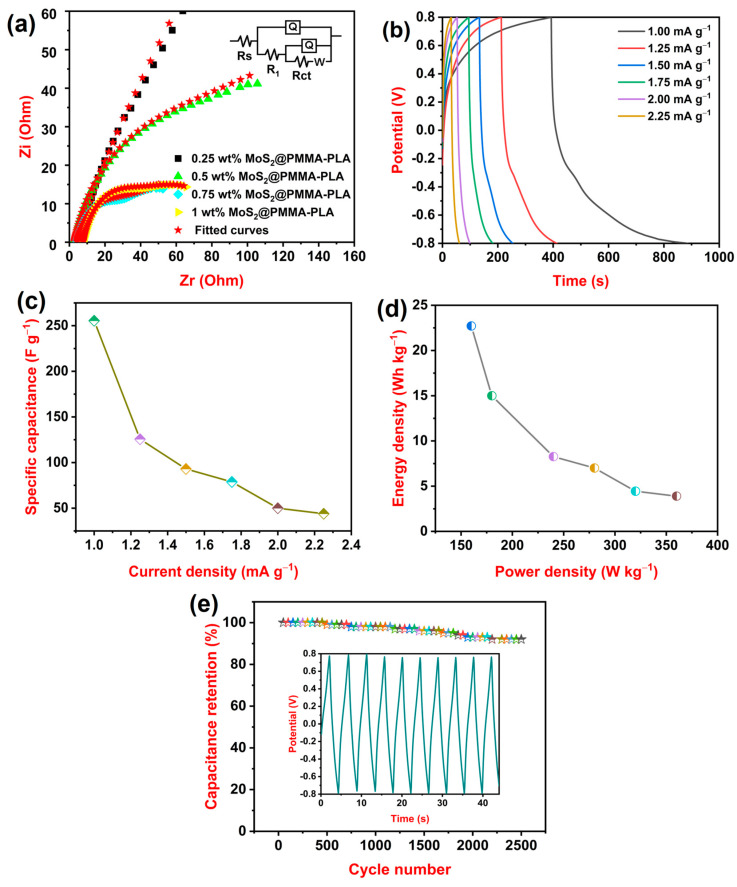
(**a**) Fitted Nyquist curves of 0.25 wt% MoS_2_@PMMA-PLA, 0.5 wt% MoS_2_@PMMA-PLA, 0.75 wt% MoS_2_@PMMA-PLA, and 1 wt% MoS_2_@PMMA-PLA with equivalent circuit diagram; (**b**) Charge–discharge curves of 0.75 wt% MoS_2_@PMMA-PLA at variable current densities; (**c**) Variation in Csp of 0.75 wt% MoS_2_@PMMA-PLA at variable current densities; (**d**) Variation in energy density concerning power density of 0.75 wt% MoS_2_@PMMA-PLA; (**e**) Life cycle of 0.75 wt% MoS_2_@PMMA-PLA at a current density of 3.00 mA g^−1^.

Figure 4a represents the fitted Nyquist plot of all the MoS_2_-loaded PMMA-PLA membranes. ZSimpWin 3.21 was used to fit all the impedance data with a matched model of equivalent circuit Rs(Q(R_1_(Q(RctW)))), where Rs—solution resistance, Q—constant phase element, R_1_—resistance component, Rct—charge transfer resistance, and W—Warburg impedance [42]. Table 2 displays the impedance data of all of the fabricated membranes. From the table, it is observed that 0.75 wt% MoS_2_@PMMA-PLA displayed the lowest values of Rs, Rct, and W and the highest value of Q among all of the fabricated membranes. As the amount of MoS_2_ increases from 0.25 wt% to 0.75 wt%, the Rs and Rct values decrease because MoS_2_ creates conductive pathways that facilitate the movement of ions and electrons, thereby reducing the overall resistance of the membrane. This improved conductivity leads to a decrease in both Rs and Rct [43]. Furthermore, the W value also decreases because MoS_2_ potentially creates a more porous or interconnected structure. This facilitates ion diffusion through the membrane, again contributing to a lower Warburg impedance [44]. As the amount of MoS_2_ increases from 0.25 wt% to 0.75 wt%, the Q value increases due to the presence of numerous active material MoS_2_. At 1 wt% amount of MoS_2_@PMMA-PLA, the Rs, Rct, and W values increase whereas the Q value decreases. This is attributed to the agglomeration of MoS_2_ at the PMMA-PLA matrix. 

### 3.7. GCD Study

Figure 4b represents GCD curves of optimized 0.75 wt% MoS_2_@PMMA-PLA membrane at different current densities 1.00, 1.25, 1.50, 1.75, 2.00, and 2.25 mA g^−1^. From the graph, it is detected that the time taken to charge–discharge is more for 1.00 mA g^−1^. As the current density decreases, the duration of the charge–discharge process increases. The rate of electrochemical reactions at the electrodes is directly proportional to the current density. When the current density drops, the reaction rates slow down, extending the time it takes for the charge to be absorbed or released from the electrode material. The Csp of the 0.75 wt% MoS_2_@PMMA-PLA membrane was calculated at different densities using Equation (2). The variation in Csp concerning current densities is presented in Figure 4c. The 0.75 wt% MoS_2_@PMMA-PLA displayed Csp values of 255.5, 125.6, 93.0, 78.7, 50.0, and 43.8 F g^−1^ at current densities of 1.00, 1.25, 1.50, 1.75, 2.00, and 2.25 mA g^−1^, respectively. It is observed that as the current density decreases, the Csp of the material increases. At lower current densities, the electrolyte ions have more time to penetrate the pores and access the entire electrode surface area. This leads to more efficient charge accumulation and utilization, resulting in higher capacitance. The energy density and power density of the 0.75 wt% MoS_2_@PMMA-PLA membrane can be calculated using Equations (3) and (4), respectively. 

Figure 4d represents the variation in energy density concerning power density. The 0.75 wt% MoS_2_@PMMA-PLA membrane showed a maximum energy density of 22.7 Wh kg^−1^ at a power density of 160 W kg^−1^. The energy density decreased to 3.9 Wh kg^−1^ at a power density of 360 W kg^−1^. A cycle study was carried out for 2500 cycles at a current density of 3.00 mA g^−1^. Figure 4e illustrates the cycle life of the 0.75 wt% MoS_2_@PMMA-PLA membrane along with the nature of the charge–discharge curves for the first 10 cycles. After prolonged 2500 cycles, the 0.75 wt% MoS_2_@PMMA-PLA membrane exhibited capacitance retention of 92%. As the cycle life increases, both the charge and discharge capacity diminish due to the deterioration of the electrode [45]. Table 3 displays a comparison study of various electrode materials composed of MoS_2_. The findings of our research are compared with other MoS_2_-based electrode materials and it is found that MoS_2_ incorporated into the PMMA-PLA matrix showed better results. Electrodes exhibit excellent capacitance retention, typically maintaining over 90% of their initial capacitance even after thousands of charge–discharge cycles. This high retention rate is crucial for applications demanding long-term reliable performance.

### 3.8. Supercapacitor Device Study

**Figure 5:**
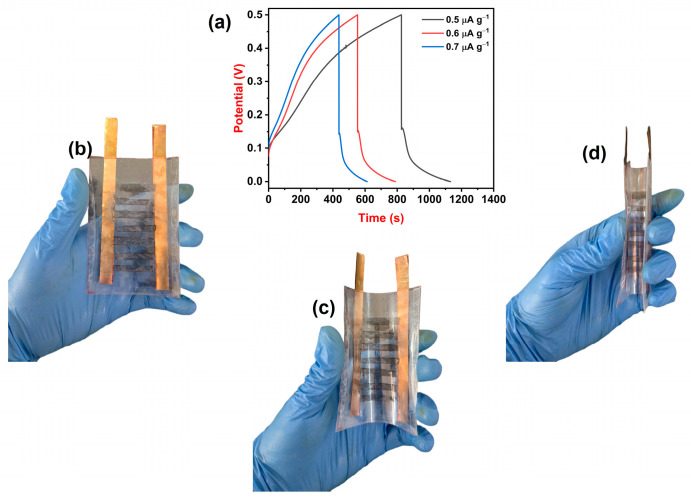
(**a**) Charge–discharge curves of the device at variable current densities. (**b**–**d**) Photo images of the device showing its flexibility at 0°, 90°, and 180°.

The designed supercapacitor device was subjected to the GCD technique to analyze its Csp at different current densities. Figure 5a represents charge–discharge curves of supercapacitor devices at current densities 0.5, 0.6, and 0.7 μA g^−1^. At a current density of 0.5 μA g^−1^, the device showed a maximum Csp of 1240 μF g^−1^ and, at a current density of 0.7 μA g^−1^, the device showed a minimum Csp of 1013 μF g^−1^. The supercapacitor device displayed a maximum energy density of 43 μWh kg^−1^ at a power density of 500 μW kg^−1^ and a minimum energy density of 35 μWh kg^−1^ at a power density of 700 μW kg^−1^. Since the device is made up of 0.75 wt% MoS_2_@PMMA-PLA membrane, it processes freestanding properties. Figure 5b–d illustrates photo images of the supercapacitor device at 0°, 90°, and 180° bending. From the figures, one can observe that the device is super flexible and that the destruction of the electrode material does not take place even after bending for 180°. Thus, supercapacitor devices of MoS_2_@PMMA-PLA membrane can be considered for the manufacture of flexible electronic devices as energy storage applications.

## 4. Conclusions

In summary, we demonstrated the electrochemical properties of MoS_2_ at PMMA-PLA membranes for supercapacitors. The FTIR technique suggested that MoS_2_ can form hydrogen bonds with PMMA-PLA blends. WCA tests showed the hydrophilicity of membranes and surface roughness. SEM analysis gave information about the formation of the microvoid structures and microcracking. UTM studies explained that MoS_2_ can act as a reinforcing filler, providing additional strength and stiffness to the material. CV and EIS techniques suggested that the 0.75 wt% MoS_2_@PMMA-PLA membrane showed better electrochemical properties. At a scan rate of 5 mV s^−1^, the 0.75 wt% MoS_2_@PMMA-PLA membrane has a Csp of 209 F g^−1^, lower findings of Rs, Rct, and W, and also higher findings of Q. From GCD tests, it can be revealed that the 0.75 wt% MoS_2_@PMMA-PLA membrane exhibited a maximum Csp of 255.5 F g^−1^ at a current density of 1.00 mA g^−1^, the maximum energy density of 22.7 Wh kg^−1^, and maximum power density of 360 W kg^−1^. The cycle study confirmed that the 0.75 wt% MoS_2_@PMMA-PLA membrane displayed 92% capacitance retention after 2500 cycles. The designed supercapacitor device showed a maximum Csp of 1240 μF g^−1^ at a current density of 0.5 μA g^−1^, a maximum energy density of 43 μWh kg^−1^, and a maximum power density of 700 μW kg^−1^. The collective findings of the current study established the real-world use of MoS_2_-based supercapacitor devices.

## Figures and Tables

**Table 1:** Mechanical properties of plain PMMA-PLA and MoS_2_-incorporated PMMA-PLA membranes.

Membranes	Tensile Strength (MPa)	Young’s Modulus (MPa)	Elongation at Break (%)
Plain PMMA-PLA	11.1	2130	1.07
0.25 wt% MoS_2_@PMMA-PLA	12.3	3761	0.628
0.5 wt% MoS_2_@PMMA-PLA	18.5	4316	0.519
0.75 wt% MoS_2_@PMMA-PLA	20.2	4463	0.338
1 wt% MoS_2_@PMMA-PLA	13.9	4167	0.426

**Table 2:** Impedance parameters of MoS_2_-incorporated PMMA-PLA membranes.

Membranes	Rs (Ω)	Q (S-s^n^)	n	R_1_ (Ω)	Q (S-s^n^)	n	Rct (Ω)	W (S-s^5^)
0.25 wt% MoS_2_@PMMA-PLA	7.404	0.00146	0.7	0.082	0.000103	0.8	489	0.000816
0.5 wt% MoS_2_@PMMA-PLA	6.329	0.00311	0.8	0.040	0.000177	0.9	317	0.000736
0.75 wt% MoS_2_@PMMA-PLA	2.852	0.00593	0.8	0.020	0.000242	0.8	106	0.000172
1 wt% MoS_2_@PMMA-PLA	4.371	0.00563	0.7	0.032	0.000178	0.8	137	0.000359

**Table 3:** Comparison study of MoS_2_ composed of different electrode materials.

Sl. no	Composition	Method	Electrolyte	Capacitance	Energy Density	Power Density	Capacitance Retention	References
1	MoS_2_/GF//AEG	Hydrothermal	3 M KCl	59 F g^−1^ @ 1 Ag^−1^	16 Wh kg^−1^	758 W kg^−1^	95 %@ 2000 cycles	[46]
2	Few layered MoS_2_	Ball milling	Organic	14.7 F g^−1^ @ 0.75 Ag^−1^	18.43 Wh kg^−1^	1125 W kg^−1^	91.2% @ 5000 cycles	[47]
3	CNT/MoS_2_	Chemical vapor deposition	PVA/H_3_PO_4_	13.16 F cm^−3^ @ 0.1 mA	1.05 mWh cm^−3^	0.46 Wcm^−3^	98 %@ 10,000 cycles	[48]
4	MoS_2_-CNT-PPY	Hydrothermal	PVA/H_2_SO_4_ /Na_2_MoO_4_	37.4 F g^−1^ @ 1 mA	3.2 Wh kg^−1^	625 W kg^−1^	94.5% @ 5000	[49]
5	AgNW-MoS_2_	Laser writing	PVA-H_2_SO_4_	27.6 mF cm^−2^ @ 0.2 V s^−1^	2.453 mWh cm^−2^	1.472 mW cm^−2^	96.4 %@ 10,000 cycles	[50]
6	MoS_2_-PEDOT:PSS	Spin coating	3 M KOH	89.44 mF cm^−2^ @ 6 mA cm^−2^	12.42 mWh cm^−2^	6043 mW cm^−2^	-	[51]
7	MoS_2_@PMMA-PLA	Solution casting	1 M H_2_SO_4_	255.5 F g^−1^ @ 1.00 mA g^−1^	22.7 Wh kg^−1^	360 W kg^−1^	92 %@ 2500 cycles	This work

## Data Availability

The data used to support the findings of this study are included in the article.
